# Risk factors for oxaliplatin-induced vascular pain in patients with colorectal cancer and comparison of the efficacy of preventive methods

**DOI:** 10.1186/s40780-018-0117-z

**Published:** 2018-08-07

**Authors:** Yukio Suga, Nana Ikeda, Manami Maeda, Angelina Yukiko Staub, Tsutomu Shimada, Miwa Yonezawa, Hironori Kitade, Hideyuki Katsura, Morihiro Okada, Junko Ishizaki, Yoshimichi Sai, Ryo Matsushita

**Affiliations:** 1 Department of Clinical Drug Informatics, Faculty of Pharmacy, Institute of Medical, Pharmaceutical & Health Science, Kanazawa University, 13-1, Takaramachi, Kanazawa, Ishikawa 920-8641 Japan; 2 Department of Pharmacy, Kanazawa University Hospital, Kanazawa University, 13-1, Takaramachi, Kanazawa, Ishikawa 920-8641 Japan; 3 Department of Pharmacy, Ishikawa Prefectural Central Hospital, 2-1, Kuratsukihigashi, Kanazawa, Ishikawa 920-8530 Japan; 4 Department of Pharmacy, Houju Memorial Hospital, 11-71, Midorigaoka, Nomi, Ishikawa 923-1226 Japan; 5 Department of Pharmacy, Komatsu Municipal Hospital, HO-60, Mukai Moto-ori-machi, Komatsu, Ishikawa 923-8560 Japan; 6 Department of Pharmacy, Japan Community Healthcare Organization Kanazawa Hospital, Ha-15, Oki-machi, Kanazawa, Ishikawa 920-8610 Japan

**Keywords:** Vascular pain, Oxaliplatin, Colorectal cancer, Preventive method, Risk factor

## Abstract

**Background:**

Vascular pain is a common adverse drug reaction in colorectal cancer patients receiving peripheral venous administration of oxaliplatin. The aim of this work was to identify risk factors for vascular pain, and to examine whether currently used treatments reduce its incidence.

**Methods:**

We conducted a multicenter retrospective study in Japanese colorectal cancer patients receiving peripheral venous administration of oxaliplatin. The effects of various treatments (administration of analgesics, addition of dexamethasone to the infusion solution for pH adjustment, dilution of the infusion solution, or use of hot gel for warming the injection site) on the incidence of vascular pain were assessed. Risk factors for vascular pain were identified by multiple logistic regression analysis.

**Results:**

One hundred and ninety patients who had received an oxaliplatin-containing regimen via a peripheral venous route were analyzed. None of the preventive methods examined significantly reduced the incidence of vascular pain. BMI (BMI < 22), clinical stage (I-III) and oxaliplatin dosage (130 mg/m^2^ versus dose reduction) were identified as independent risk factors for development of vascular pain. The incidence of oxaliplatin-induced vascular pain was significantly higher in patients who had two or more risk factors.

**Conclusions:**

BMI, clinical stage and oxaliplatin dosage were identified as independent predictive markers for oxaliplatin-induced vascular pain. Existing treatments for vascular pain are not effective in reducing its incidence.

## Background

Oxaliplatin (L-OHP) is a third-generation platinum analog that is mainly used for treatment of advanced colorectal cancer [1] and adjuvant chemotherapy [2]. Although peripheral neuropathy is a major adverse drug reaction in patients receiving L-OHP [3–5], mild to moderate vascular pain originating around the injection site during peripheral intravenous administration of L-OHP is also a significant problem. The mechanism of L-OHP-induced vascular pain is poorly understood. Also, risk factors and preventive methods have not yet been adequately evaluated.

Propofol has also been reported to cause vascular pain, like L-OHP [6–8]. It was reported that preadministration of fentanyl or remifentanyl (opioid analgesics) tended to reduce the incidence of propofol-induced vascular pain [6]. Several methods for preventing or relieving L-OHP-induced vascular pain have been reported in clinical studies, but only small numbers of patients were involved [9–11]. Shiotsuka et al. reported that addition of 3.3 mg of dexamethasone to the infusion solution reduced the numerical rating scale (NRS) score of vascular pain induced by peripheral administration of L-OHP [10]. Hibi et al. reported that warming the injection site with a hot gel was effective for managing L-OHP-induced vascular pain [11]. Studies with larger numbers of patients are necessary to confirm and compare the efficacy of these methods.

It is important to elucidate the risk factors for adverse drug reactions in order to develop effective preventive methods. For example, the use of epirubicin liquid preparation was identified as a risk factor for phlebitis [12], and we have shown that the incidence of epirubicin-induced severe phlebitis could be significantly reduced by employing a lyophilized formulation [13]. However, the risk factors for L-OHP-induced vascular pain are not clear.

The present multicenter retrospective study was conducted in order to determine whether currently used treatments are effective to reduce the incidence of vascular pain in colorectal cancer patients receiving peripheral venous administration of L-OHP. We also investigated the risk factors for L-OHP-induced vascular pain.

## Methods

### Patients

The present study was conducted in accordance with the Declaration of Helsinki and its amendments, and the protocol was approved by the Ethics Committee of Kanazawa University (approval no. 1462), as well as by the ethics committees of all participating hospitals, which were Ishikawa Prefectural Central Hospital, Houju Memorial Hospital, Komatsu Municipal Hospital, and Japan Community Healthcare Organization Kanazawa Hospital (approval no. 507, 14–2, 10 and 13–17-00, respectively). Patients who had received peripheral venous administration of L-OHP during April 2011 to March 2014 were collected from 5 hospitals in Ishikawa prefecture. We excluded patients who were fitted with a peripheral venous catheter port, or for whom any of the clinical data required in the study protocol were missing.

### Study protocol

Data were collected from the nursing records and computerized medical records. Clinical data included age, gender, previous history of chemotherapy, dosage and infusion time of L-OHP, development of venous pain, concomitant drug used for analgesia, addition of dexamethasone, volume of infusion solution, and use of hot gel. For patients with a history of L-OHP-induced vascular pain, data were selected for the first cycle in which vascular pain occurred, while for patients who did not encounter vascular pain in any cycle, we selected data from the first cycle of L-OHP.

We assessed the incidence of L-OHP-induced vascular pain in patients classified according to use of analgesics, addition of dexamethasone, volume of infusion solution, or use of hot gel to warm the injection site, compared to patients who did not receive that treatment. Univariate analysis and multiple logistic regression analysis were conducted to identify independent risk factors associated with L-OHP-induced vascular pain. Based on the multiple logistic regression analysis, we also examined the relationship between the number of risk factors and the incidence of L-OHP-induced vascular pain.

### Statistical analyses

The incidence of L-OHP-induced vascular pain in patients who received each preventive therapy was compared with the incidence in those who did not receive it by applying Fisher’s exact test. The relationship between the number of risk factors and the incidence of L-OHP-induced vascular pain was also analyzed by means of Fisher’s exact test. To identify risk factors associated with L-OHP-induced vascular pain, multiple logistic regression analysis was performed. Factors for which *P* < 0.20 in univariate analysis were selected for multiple logistic regression analysis.

Data were analyzed using IBM SPSS Version 19 and *P* values of < 0.05 were considered statistically significant.

## Results

### Patient characteristics

One hundred and ninety colorectal cancer patients who had received peripheral venous administration of L-OHP were studied. The total number of infusions of L-OHP was 1, 264. There were 117 males and 73 female with a mean age of 64.2 ± 11.3 years (Table 1). The dosage of dexamethasone mixed with infusion solution for prevention of vascular pain was 1.65 mg in all patients who received this preventive method. Use of analgesic was defined as routine administration of nonsteroidal anti-inflammatory drugs (NSAIDs) and/or opioids. NSAIDs administered to patients in this study were loxoprofen, diclofenac, acetaminophen, and celecoxib, and opioids used in this study were morphine sulfate, oxycodone hydrochloride and fentanyl patch. As the number of patients given analgesics was very small (Table 2), the preventive effect on vascular pain could not be analyzed precisely.

**Table 1:** Patient characteristics

Number of patients (number)			190
Number of infusions (number)			1264
Gender			
		Male	117
		Female	73
Age (year)	means ± SD		64.2 ± 11.3
BMI (kg/m^2^)	means ± SD		22.1 ± 3.2
History of chemotherapy (number)			
		1st	173
		2nd	17
Clinical stage (number)			
		I	2
		II	15
		III	92
		IV	81
L-OHP dosage (number)			
		130 mg/m^2^	92
		< 130 mg/m^2^	98

*SD* standard deviation

**Table 2:** Univariate analysis of risk factors for oxaliplatin-induced vascular pain

		Vascular pain (+)	Vascular pain (−)	Odds ratio	95%CI	*P* value
	Incidence (%)	*n* = 124	*n* = 66			
Gender						
Female	64.4	47	26	0.94	0.51–1.73	0.876
Male	65.8	77	40			
Age						
≧ 70	62.1	41	25	0.81	0.43–1.51	0.526
< 70	66.9	83	41			
BMI						
≧ 22	58.2	53	38	0.55	0.30–1.01	0.067
< 22	71.7	71	28			
Clinical stage						
IV	56.8	46	35	0.52	0.29–0.96	0.045
I-III	71.6	78	31			
History of chemotherapy						
2nd	41.2	7	10	0.34	0.12–0.93	0.035
1st	67.6	117	56			
L-OHP dosage						
< 130 mg/m^2^	57.1	56	42	0.47	0.25–0.87	0.022
130 mg/m^2^	73.9	68	24			
Solution volume mixed with oxaliplatin						
500 mL	67.2	41	20	1.14	0.60–2.17	0.746
250 mL	64.3	83	46			
Warming the injection site						
Yes	63.3	81	47	0.76	0.40–1.46	0.516
No	69.4	43	19			
Analgesics						
Yes	56.3	9	7	0.66	0.23–1.86	0.425
No	66.1	115	59			
Opioids						
Yes	62.5	5	3	0.88	0.20–3.81	1.000
No	65.4	119	63			
Non-opioids						
Yes	54.5	6	5	0.62	0.18–2.12	0.519
No	65.9	118	61			
pH adjustment (addition of dexamethasone to infusion solution mixed with oxaliplatin)						
Yes	78.3	18	5	2.07	0.73–5.86	0.242
No	63.5	106	61			

*CI* confidence interval

### Incidence of L-OHP-induced vascular pain in patients with or without use of analgesics, addition with dexamethasone, dilution of infusion solution, or use of hot gel

**Fig. 1:**
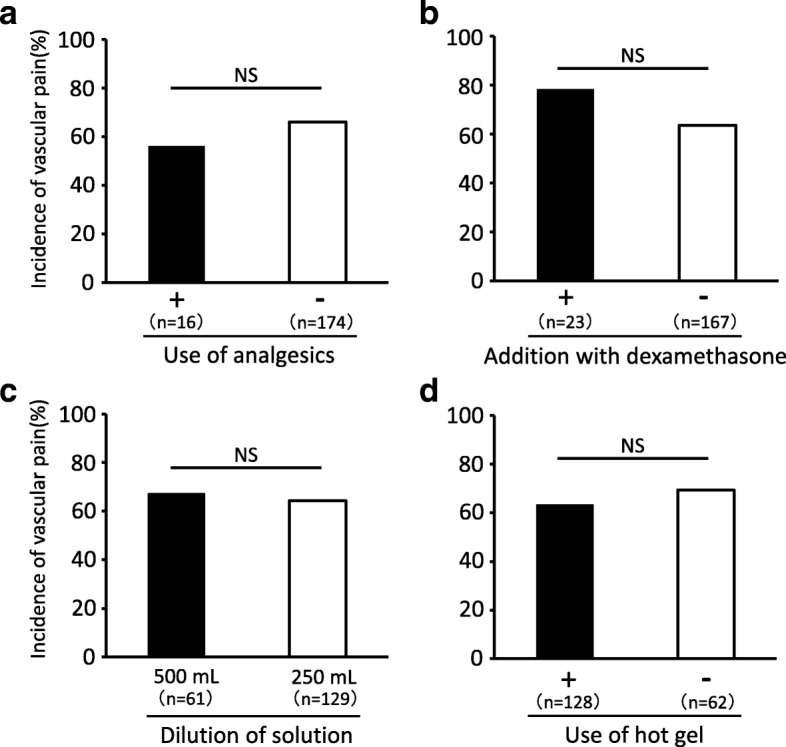
The incidence of L-OHP-induced vascular pain in patients with or without use of analgesics (**a**), addition of dexamethasone to adjust the pH of the infusion solution (**b**), dilution of the infusion solution (**c**) and use of hot gel to warm the injection site (**d**). NS: not significant (Fischer’s exact test)

The incidences of L-OHP-induced vascular pain were 56.3% in patients receiving analgesics, 78.3% in those given dexamethasone, 67.2% in those receiving diluted infusion solution and 63.3% in those who were given hot gel to warm the injection site. None of these methods had a significant benefit in terms of reducing the incidence of vascular pain (Fig. 1).

### Univariate and multivariable analyses of risk factors for L-OHP-induced vascular pain

In univariate analysis, the factors with *P* values < 0.20 were BMI, clinical stage, history of chemotherapy, and L-OHP dosage (Table 2). These factors were included in the multivariable analysis, which indicated that BMI, clinical stage and L-OHP dosage were independent risk factors (Table 3).

**Table 3:** Multivariate analysis of risk factors for oxaliplatin-induced vascular pain

	Adjusted odds ratio	95%CI	*P* value
BMI			
≧ 22	0.48	0.26–0.91	0.025
< 22			
Clinical stage			
IV	0.52	0.27–0.97	0.041
I – III			
History of chemotherapy			
2nd	0.43	0.15–1.28	0.129
1st			
L-OHP dosage			
< 130 mg/m^2^	0.51	0.27–0.98	0.042
130 mg/m^2^			

*CI* confidence interval

### Relationship between number of risk factors and incidence of L-OHP-induced vascular pain

**Fig. 2:**
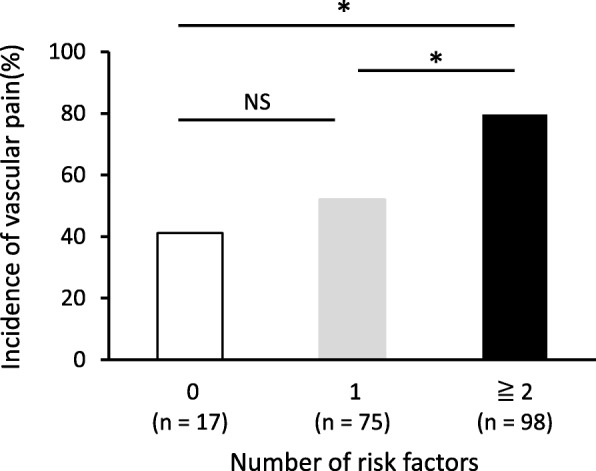
The incidence of oxaliplatin-induced vascular pain in patients according to the number of risk factors *P < 0.05 (Fischer’s exact test)

The incidences of L-OHP-induced vascular pain were 41.2% in patients who had no risk factor, 52.0% in those who had one risk factor and 79.6% in those who had two or more risk factors. Patients with two or more risk factors showed significantly increased incidence of vascular pain compared to those without risk factors (Fig. 2).

## Discussion

In this study, we identified BMI (BMI < 22), clinical stage (I-III) and L-OHP dosage (130 mg/m^2^ versus dose reduction) as independent risk factors of L-OHP-induced vascular pain. Generally, treatment to prevent vascular pain is given to patients who have already experienced vascular pain during a previous administration. Our results suggest that patients with two or more two risk factors might be at particular risk of vascular pain even in the first course.

Unfortunately, all the preventive methods examined in this study, including addition of dexamethasone to adjust the pH of the infusion solution and dilution of infusion solution, proved to be ineffective in reducing the incidence of vascular pain (Fig. 1). Although the pH or osmotic pressure of the infusion solution was proposed to cause vascular pain, our results indicate that this is unlikely. The pH of infusion solution containing 130 mg/m^2^ L-OHP in Japanese patients of average physique was 4.81 after dilution with 250 mL of 5% glucose solution and 4.82 after dilution with 500 mL. Addition of 3.3 mg dexamethasone elevated the pH of L-OHP infusion solution to 7.18 in 250 mL of 5% glucose solution and to 6.84 in 500 mL [14]. Patients in this study received 1.65 mg of dexamethasone, which should have been sufficient to elevate the pH of the L-OHP infusion solution, but the incidence of L-OHP-induced vascular pain was not reduced. On the other hand, the osmotic pressure of L-OHP infusion solution was 318 mEq/L after dilution with 250 mL of 5% glucose solution and 307 mEq/L after dilution with 500 mL [14], while the normal plasma osmotic pressure is approximately 300 mEq/L. These results suggest that neither pH nor osmotic pressure of the L-OHP infusion solution was related to the induction of vascular pain. It was reported that addition of dexamethasone to L-OHP infusion solution and heating the infusion site had a preventive or ameliorating effect on vascular pain [9, 10]. In this study, however, we assessed only whether or not the treatments altered the incidence of vascular pain, as the available data did not allow us to determine whether they were effective in reducing the severity of pain. Nagao et al. have reported that pretreatment with fast-acting oxycodone reduced vascular pain induced by oxaliplatin via a peripheral vein [15]. However, in our study, treatment with analgesics including opioids and/or NSAIDs did not reduce the incidence of vascular pain (Fig. 1). One of the differences between the two studies was the administration time of analgesics. Nagao et al. administered fast-acting oxycodone to patients 45 min before the administration of L-OHP. On the other hand, since the purpose of analgesics, including opioids and NSAIDs, in the patients in our study was to relieve cancer-related pain, the administration times might not have been appropriate to prevent vascular pain, resulting in the apparent ineffectiveness of analgesics. Carefully timed pretreatment with fast-acting oxycodone could be a useful option for management of L-OHP-induced vascular pain.

We identified three independent risk factors for L-OHP-induced vascular pain: L-OHP dosage, BMI and clinical stage (Table 3). Takagi et al. have already reported that L-OHP dosages of more than 175 mg/body are significantly related the development of vascular pain [16], suggesting that vascular pain is more likely to occur at higher L-OHP dosages. In general, L-OHP is administered over a period of 2 h. Lengthening the administration time of L-OHP would reduce the L-OHP exposure per hour, and thus might be effective to prevent vascular pain. However, it would be necessary to confirm that this does not affect the efficacy or safety of L-OHP-containing regimens. Our multivariable analysis also indicated that clinical stage I-III and BMI < 22 were independent risk factors for vascular pain. However, although vascular pain, clinical stage and BMI were all identified as risk factors by multivariate analysis, there is no clear evidence to indicate the nature of the relationship among them, or what the underlying mechanism might be. There is a report that acute cold sensitivity is one of the symptoms of acute peripheral neuropathy induced by L-OHP [5]. The acute cold sensitivity appeared within a few hours after starting infusion of oxaliplatin, at the same time as vascular pain, so a similar mechanism might be involved. Transient receptor potential ankyrin 1 (TRPA1) is activated by noxious cold stimulation and various irritants, such as reactive oxygen species (ROS) [17], and is involved in acute cold sensitivity in mice [18]. On the other hand, Ando et al. reported that TRPA1 was related to the development of vascular pain induced by propofol in a rat model [19]. However, it is not known whether or not TRPA1 is related to L-OHP-induced vascular pain. Further research will be needed to elucidate this point and to uncover the relationship between vascular pain and BMI or clinical stage.

Our results indicate that BMI, clinical stage and L-OHP dosage are useful predictive markers for L-OHP-induced vascular pain in patients with colorectal cancer. None of the preventive methods examined was effective in reducing the incidence of vascular pain, but we could not establish whether they reduced the degree of pain. Since the incidence of vascular pain was higher in patients with two or more risk factors, it may therefore be worthwhile to administer preventive treatment to high-risk individuals even upon first administration of L-OHP. Further studies are needed to elucidate the mechanism of vascular pain in order to develop effective preventive methods, as well as to compare the efficacy of the various existing treatments in ameliorating pain.

## Conclusions

The results of this retrospective study indicate that L-OHP dose (130 mg/m^2^ versus dose reduction), clinical stage I-III and BMI < 22 are risk factors for L-OHP-induced vascular pain in patients with colorectal cancer. These findings could be useful to assess the risk of vascular pain in clinical settings. Based on the results of this study together with previous findings, we suggest that the best option for management of L-OHP-induced vascular pain may be carefully timed pretreatment with fast-acting oxycodone.
